# Prediction of *in vivo* hip contact forces during common activities of daily living using a segment-based musculoskeletal model

**DOI:** 10.3389/fbioe.2022.995279

**Published:** 2022-12-15

**Authors:** Pouya Amiri, Anthony M. J. Bull

**Affiliations:** Department of Bioengineering, Imperial College London, London, United Kingdom

**Keywords:** musculoskeletal modelling, hip contact force, muscle force, optimization, activities of daily living, electromyography, segment-based model

## Abstract

**Background:** Quantifying *in vivo* hip muscle and contact forces during activities of daily living (ADL) provides valuable information for diagnosis and treatment of hip-related disorders. The objective of this study was to utilize Freebody, a segment-based musculoskeletal model, for the prediction of hip contact forces using a novel objective function during seven common ADLs and validate its performance against the publicly available HIP98 dataset.

**Methods:** Marker data, ground reaction forces, and hip contact forces during slow, normal, and fast walking, stair ascent and descent, and standing up and sitting down were extracted for 3 subjects from the HIP98 dataset. A musculoskeletal anatomical dataset was scaled to match the dimensions of each subject, and muscle and hip contact forces were estimated by minimizing a novel objective function, which was the summation of the muscle stresses squared and body weight-normalised hip contact force. The accuracy of predictions were quantified using several metrics, and muscle forces were qualitatively compared to experimental EMGs in the literature.

**Results:** FreeBody predicted the hip contact forces during the ADLs with encouraging accuracy: The root mean squared error of predictions were 44.0 ± 8.5, 47.4 ± 6.5, and 59.8 ± 7.1% BW during slow, normal, and fast walking, 44.2 ± 16.8% and 53.3 ± 12.2% BW for stair ascent and descent, and 31.8 ± 8.2% and 17.1 ± 5.0% BW for standing up and sitting down, respectively. The error in prediction of peak hip contact forces were 14–18%, 24–28%, 17–35% for slow, normal, and fast walking, 7–25% and 15–32% in stair ascent and descent, and around 10% for standing up and sitting down. The coefficient of determination was larger than 0.90 in all activities except in standing up (0.86 ± 0.08).

**Conclusion:** This study has implemented a novel objective function in a segment-based musculoskeletal model, FreeBody, for the prediction of hip contact forces during a large range of ADLs. The model outputs compare favourably for all ADLs and are the best in standing up and sitting down, while muscle activation patterns are consistent with experimental EMGs from literature. This new objective function addresses one of the major limitations associated with musculoskeletal models in the literature, namely the high non-physiological predicted hip joint contact forces.

## 1 Introduction

Quantifying *in vivo* lower limb muscle and articular contact forces during common activities of daily living (ADL) provides valuable information that can be used to understand normal and pathological human movement. For example, high articular joint contact forces are implicated in the generation and progression of osteoarthritis (OA) (Felson 2013), and can be used to design implants for total hip (Heller et al., 2011) and knee replacement surgeries (Smith et al., 2016). Muscle forces also offer insight into the neuromuscular strategies in healthy and pathological movement, thus, provide potential targets to plan and evaluate rehabilitation programs (Rane and Bull 2016; Xu et al., 2019). *In vivo* musculoskeletal (MSK) loads have been investigated widely in walking as it is the most common ADL. However, such practice fails to provide a holistic view of the mechanical environment of the joints and/or neuromuscular adaptations that happen as a result of a MSK disorder. For example, patients with early-stage knee OA first experience knee pain in stair ascending (Hensor et al., 2015), or patients with mild-to-moderate hip OA tend to apply lower ground reaction forces (GRF) on their affected limb during sit-to-stand (Eitzen et al., 2014). As such, investigating these activities may offer biomarkers to detect the disease at its early stages, when the interventions are most effective. Therefore, effective diagnosis/monitoring/management of diverse MSK disorders requires quantifying internal musculoskeletal loads during different ADLs.

Ethical considerations and technical challenges in direct measurement of *in vivo* lower limb forces have led researchers to develop MSK models that can be utilized to estimate muscle and articular contact forces during gait. The availability of experimental knee and hip joint contact forces (Bergmann et al., 2001; Fregly et al., 2012), measured from patients implanted with instrumented prostheses, has provided the opportunity to validate the performance of such models. Since muscle forces are the main contributors to joint contact forces (Erdemir et al., 2007), correct prediction of the joint contact forces also provides an indirect validation of the estimated muscle forces that can be used to study healthy and pathological neuromuscular coordination during ADLs. The “Grand challenge competition to predict *in vivo* knee loads” has provided a complete set of data to validate MSK models for the prediction of knee articular contact forces (Fregly et al., 2012) and several models have demonstrated encouraging accuracy in the estimation of *in vivo* knee loads in different ADLs (Kinney et al., 2013; Manal and Buchanan 2013; Guess et al., 2014; Ding et al., 2016). The HIP98 dataset similarly provides synchronous gait and *in vivo* hip contact loads (Bergmann et al., 2001), and has been used in several MSK models, which generated promising results for the predictions of hip contact forces (Heller et al., 2001; Stansfield et al., 2003; Modenese et al., 2011; Modenese and Phillips 2012; Moissenet et al., 2015; Mathai and Gupta 2019; Weinhandl and Bennett 2019). However, the validation has been performed for a subset of the available activities in HIP98, mostly walking at different speeds, and stair ascending.

The majority of the MSK models utilize a joint-based approach, meaning that the segment movements are constrained by the defined degrees of freedom (DOF) of the joints. These models do not account for variation in contact geometry (which may happen due to different loading profiles or pathology) at the joint level. In addition, using joint-based MSK models, muscle and articular contact force estimation is generally performed in two successive steps, because the equations of motion are posed with respect to joints, thus articular contact forces do not emerge in the equations. OpenSim (Delp et al., 2007) and Anybody (Damsgaard et al., 2006) software packages are notable examples of this joint-based approach. Almost all the previous MSK models that used the HIP98 dataset were developed using a joint-based approach, with a variety of MSK geometries (Heller et al., 2001; Stansfield et al., 2003; Modenese et al., 2011; Modenese and Phillips 2012; Mathai and Gupta 2019; Weinhandl and Bennett 2019). Heller et al. (2001) and Stansfield et al. (2003) developed their models using anatomical datasets from Brand et al. (1982), whereas others used OpenSim software to implement several MSK anatomical geometries (Modenese et al., 2011; Mathai and Gupta 2019; Weinhandl and Bennett 2019). Modenese et al. (2011) developed the London Lower Limb Model (LLM) using the methodology taken from Klein Horsman et al., 2007. Mathai and Gupta (2019) and Weinhandl and Bennett (2019) later compared the performance of LLLM with other MSK geometries within OpenSim, including gait2392 (Delp et al., 1990), the Arnold Lower Limb Model (ALLM) (Arnold et al., 2010), and a hip specific model, the hip2372 (Shelburne et al., 2010), and reported that LLLM produced the best accuracy for the predictions of the hip contact forces.

Segment-based MSK models, on the other hand, allow 6 DOF and pose the equations of motion for each segment, which result in inclusion and simultaneous estimation of *in vivo* articular contact forces, and muscle and tendon forces (Cleather and Bull 2015). This is a strength of segment-based models that allows articular contact forces to be directly included in the objective function of muscle force sharing to manipulate muscle co-contractions. FreeBody is a segment-based 3D MSK modelling software (Cleather and Bull 2015), which has been validated for the prediction of *in vivo* knee contact forces during different ADLs, generating encouraging results (Ding et al., 2016). The validated model has provided the means to investigate the efficacy of clinical interventions that reduced knee contact forces during gait (Rane and Bull 2016; Azmi et al., 2018; Xu et al., 2019). However, FreeBody has not been validated for the prediction of hip joint contact forces. This is necessary to assess the efficacy of interventions, aimed at reducing hip contact forces in pathological gait, for example, in patients with hip OA or in transfemoral amputees, whose overload of hip muscles (due to loss of ankle and knee joints) lead to elevated hip contact forces (Toderita et al., 2021).

Therefore, the objective of this study was to utilize Freebody, a segment-based musculoskeletal model, for the prediction of hip contact forces using a novel objective function during seven common ADLs and validate its performance against the publicly available HIP98 dataset, and compare the performance of the model against others in the literature, which have previously reported their predictions of the HIP98 dataset for a subset of ADLs.

## 2 Materials and methods

### 2.1 Experimental data

The experimental data used in the current study were taken from the publicly available HIP98 dataset, which contains ADL data (marker displacement and ground reaction forces (GRF)) and simultaneous hip joint contact forces of 4 patients, who underwent hip replacement surgery (Bergmann et al., 2001). The data during the most common ADLs, including slow, normal, and fast walking (WS, WN, WF), stair ascending and descending (SU and SD), and standing up and sitting down (CU and CD), were extracted for 3 subjects from the dataset (subject IBL was not included in the study, since she did not have any data for 3 out of the 7 ADLs.). Table 1 shows the included subjects’ demographics and the number of trials available for each of the investigated ADLs. Marker trajectories, GRF, and simultaneous hip contact forces during dynamic trials were extracted and the first 0.1 s of standing trials (2–1-2 legs in (Bergmann et al., 2001)) was used as the calibration trial for further processing.

**TABLE 1:** Subjects’ demographics and the number of trials for each activity. WN, WS, WF, SU, SD, CD, and CU stand for normal walking, slow walking, fast walking, stair ascent, stair descent, sitting down and standing up, respectively.

Subject	Age (y)	Weight (N)	Height (cm)	Sex	Number of trials						
					WN	WS	WF	SU	SD	CD	CU
HSR	55	860	174	M	9	1	6	7	5	5	5
KWR	61	702	165	M	9	6	6	7	7	5	5
PFL	52	980	175	M	7	6	5	3	1	5	5

### 2.2 Musculoskeletal model

FreeBody, which is a 3D segment-based MSK model, was used in this study (Cleather and Bull 2015). FreeBody model comprises 5 segments: foot, shank, thigh, patella, and pelvis that are articulated through 4 joints: ankle, tibiofemoral, patellofemoral, and hip. All segments are allowed to move freely, except the hip in this version of the model, since the instrumented replacement hip allows only 3 rotational DOF. Muscle, ligaments, and contact forces act upon the segments and move them with respect to each other. The MSK model contains 163 line force elements, which represent 38 muscles in the lower limb, according to the methodology proposed by Klein Horsman et al., 2007. The attachments sites of these muscle force elements, including origin, *via* and insertion points, as well as joint rotation centers and bony anatomical landmarks are defined in the MSK model (Ding et al., 2019). Two cylindrical wrapping surfaces are utilized to represent iliopsoas and medial gastrocnemius muscle elements actions along superior pubic ramus of pelvis and femoral condyles, respectively. Hip contact forces are assumed to be applied to the center of a sphere (representing the femoral head) and the tibiofemoral joint is split into medial and lateral compartments, the centers of which are the application points of the contact forces. The maximum force potential of each muscle is obtained by multiplying the physiological cross-sectional area of the muscle by an assumed maximum stress of 31.39 N/cm^2^ (Yamaguchi 2005).

To define the MSK anatomy of each subject in this study, a MSK anatomical dataset of a healthy individual (height = 1.80 m, mass = 70.0 kg, sex = male, age = 25 years), obtained from high resolution MRI images of lower limb (from pelvis to the most distal part of the foot) was scaled to match the dimension of the subject. The scaling factors for foot, shank, and thigh were obtained by comparing the subjects’ limb lengths in HIP98 and the healthy anatomical dataset. For the pelvis, the scaling factor was obtained by comparing the distance between the hip joint centers of the anatomical dataset and the subject’s hip joint centers, calculated from the calibration trial of HIP98 dataset. To apply the scaling factors, the muscle attachment sites, joint rotation centers, articular contact points, and wrapping surfaces in the original healthy dataset were multiplied by the scaling factors of the segment that they were attached to.

#### 2.3 Inverse kinematics and inverse dynamics

Available experimental marker data for each subject were filtered using a fourth order Butterworth low pass filter with a corner frequency of 10 Hz. Virtual markers were attached to the corresponding landmarks in the scaled MSK model. To determine the kinematics of movement (i.e. joint angles) during each trial, for each time frame an optimization problem was solved that minimized the root mean squared error (RMSE) of the distance between the experimental and virtual markers (Lu and O’Connor, 1999). The kinematic data were then utilized within the equation of motion of the MSK system (based on a wrench formulation developed by Dumas et al. (2004)) to determine net forces and moments, acting upon joints of lower limbs. The inertial parameters and position of the center of mass of the segments, required for the inverse dynamic calculations were obtained using regression equations proposed by de Leva (1996).

### 2.4 Muscle and joint contact force estimation

Actuation redundancy in the MSK system results in an indeterminate problem, where infinite combination of muscle forces can produce an observed movement (kinematics and external force) (Erdemir et al., 2007). To resolve this problem, the most common method is to solve a constrained static optimization problem that minimizes the sum of muscle forces or stresses (with a certain power), which must satisfy the equations of motion of the MSK system (i.e. the internal forces of muscles must balance the external moments found from inverse dynamics). In the current study, FreeBody used an objective function, which was a combination of the sum of squared normalized muscle forces (stresses) and the magnitude of hip contact force. The first term of the objective function, the sum of squared muscle stresses, has been demonstrated to generate realistic activation patterns for lower limb muscles; however, while it generates reasonable estimates of knee (Knarr and Higginson 2015) and hip (Modenese et al., 2011) contact forces, it has been demonstrated to overestimate the hip contact forces (Modenese et al., 2011; Modenese et al., 2013). Thus, taking advantage of the segment-based formulation of FreeBody (where muscle and articular contact forces are estimated simultaneously), the second term, body weight normalized magnitude of hip contact force, was included in the cost function to generate more physiological muscle and hip contact forces. This approach was previously demonstrated to improve the prediction of knee contact forces (DeMers et al., 2014). Furthermore, FreeBody uses a non-linear solver (the fmincon function within MATLAB) to solve the optimization problem, whose solution is heavily dependent on initial conditions and is not guaranteed to converge to the global minimum. The inclusion of the magnitude of the hip contact force drives the solution toward smaller values, closer to the actual minimum. The problem is formulated as (1):

$$\begin{array}{c} \min J=\overset{163}{\underset{i=1}{\sum }}{\left(\frac{F_{i}}{F_{imax}}\right)}^{2}+\alpha \;\frac{\left|HCF\right|}{BW}\\ {0\leq F}_{i}\leq F_{imax}\;,\;i=1,\ldots ,163\\ \begin{array}{c} subject\;to:\\ \left[\begin{array}{c} \overset{L}{\underset{l=1}{\sum }}F_{l}∙n_{lm}-\overset{K}{\underset{k=1}{\sum }}F_{k}∙n_{k\left(m-1\right)}+J_{m}-J_{m-1}\\ \overset{L}{\underset{l=1}{\sum }}F_{l}∙n_{lm}\times r_{lm}-\overset{K}{\underset{k=1}{\sum }}F_{k}∙n_{k\left(m-1\right)}\times r_{k\left(m-1\right)}-d_{m}\times J_{m-1} \end{array}\right] \end{array} \end{array}=\left[\begin{array}{cc} M_{m}E_{3\times 3} & 0_{3\times 3}\\ M_{m}\tilde{c_{m}} & I_{m} \end{array}\right]\left[\begin{array}{c} a_{m}-g\\ {\ddot{\theta }}_{m} \end{array}\right]+\left[\begin{array}{c} 0_{3\times 1}\\ {\dot{\theta }}_{m}\times I_{m}{\dot{\theta }}_{m} \end{array}\right]$$ (1)

where the matrix calculations contain the equations of motion of foot, shank, thigh, and patella with the following:

$F_{i}$ : force of the *ith* muscle element (*i* = 1, … , 163)

$F_{imax}$ : maximum force potential of the *ith* muscle element

$\left|HCF\right|$ : magnitude of 3D vector of hip contact force

BW : body weight

α : weighting factor for the hip contact force magnitude

m : number of the segment (numbering from distal to proximal, m = 1, … , 5)

*L* and *K*: The number of proximal and distal muscle elements acting on segment *m*, respectively.

$n_{lm}$ , $n_{l\left(m-1\right)}$ : unit vector representing the line of action of proximal and distal muscle elements acting on segment m , respectively

${J_{m},J}_{m-1}$ : proximal and distal joint articular contact forces applied to segment m , respectively

$r_{lm},r_{l\left(m-1\right)}$ : moment arm vector of the proximal and distal muscle elements with respect to center of rotation of the joint at the proximal end of the segment *m*

***dm:***

vector from the proximal to the distal joint rotation centers of segment *m*

***c*** ***m:***

vector from the proximal joint rotation center to the center of mass of segment *m*

$\tilde{c_{m}}$ : skew symmetric matrix of *c*_*m*_

$E_{3\times 3}$ : 3 by 3 identity matrix

$I_{m}$ and $M_{m}$ : inertia matrix and mass of the segment *m*

${\dot{\theta }}_{m}$ and ${\ddot{\theta }}_{m}$ : angular velocity and acceleration of segment *m*

**FIGURE 1:**
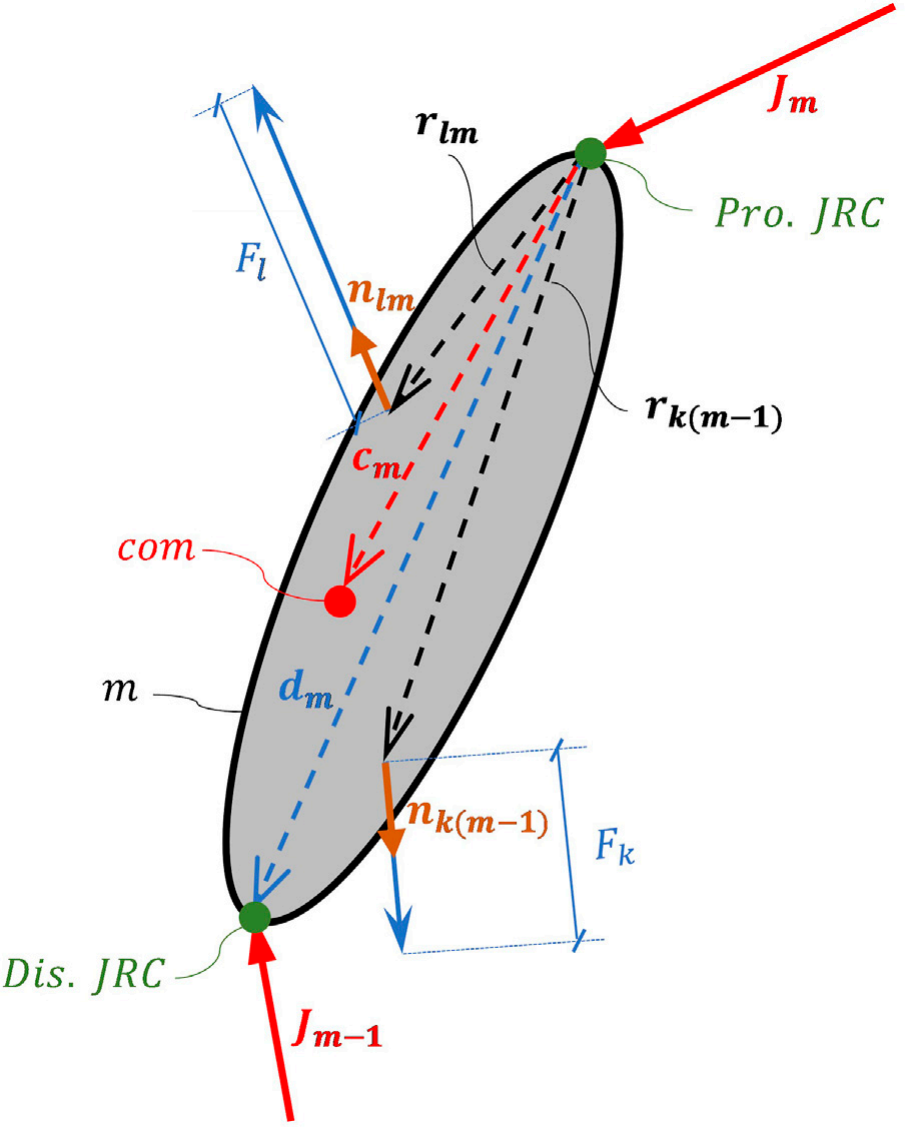
Diagram demonstrating segment m and the symbols used in equation 1. Pro. JRC and Dis. JRC stand for proximal and distal joint Rotation Centers, respectively. com shows the center of mass of the segment.

Figure 1 shows a schematic, representing the quantities used in Eq. 1.

Through a sensitivity analysis, it was found that the most suitable value for the weighting factor ( α ) for hip contact force was 1 for all ADLs and subjects, since the analysis demonstrated that larger weighting factors resulted in some muscle forces being forced to very small non-physiological values. In addition, the maximum force potentials of the muscles were modified to account for higher age of the HIP98 dataset (age = 55, 61, and 52 years) compared to the FreeBody MSK anatomical dataset (age = 25 years). Lower limb muscle strengths have been reported to reduce with ageing: ankle muscle strength was 25% lower between 32 and 72 year-old (Kent-Braun and Ng 1999), knee extensors and flexors strengths were both around 25% lower at 70.7 years compared to 24.5 years (Overend et al., 1992), hip abductors and adductors were 34% and 24% lower, respectively, between 23 and 74 years (Johnson et al., 2004), and hip extensors strengths were 26% smaller between two cohorts with average age of 26 and 67 years (Palmer et al., 2017). In addition, hip abductors (gluteus medius, gluteus minimus and tensor fasciae latae, which are the main contributors to hip contact forces (Correa et al., 2010)) have been shown to be 30% weaker than age-matched controls 12 months after total hip replacement surgery (all our subjects’ data were collected between 11 and 14 months post-operation, Table 1) (Murray et al., 1979). Therefore, ankle and knee muscles’ maximum force potentials were reduced by 30%, hip adductors and extensors’ maximum force potentials were reduced by 25%, and the hip abductors’ maximum forces were reduced by 50% (to account for both ageing and the surgery).

### 2.5 Evaluation of model predictions

To evaluate the performance of the MSK model for the prediction of *in vivo* hip contact forces, the following variables were obtained:

1) Error in the prediction of the peaks of total hip contact force: the errors were obtained by finding the absolute difference between the predicted and measured peak hip contact forces, normalized to the measured peak. In walking and stair trials, generally two peaks existed, during the loading response and the push-off; however, for standing up and sitting down trials only one peak was observed.
2) Error in the timing of the predicted and measured peak hip contact forces in terms of percentage of the activity cycle (absolute value was obtained.)
3) Body weight normalized RMSE between the measured and predicted total hip contact forces, as a global measure of the goodness of the fit.
4) Coefficient of determination (*R* ^2^) between the measured and predicted total hip contact force waveforms.
5) Muscle activation (i.e. muscle force normalized to its maximum force potential) patterns were also qualitatively compared to the experimental EMGs from the literature.

## 3 Results

**FIGURE 2:**
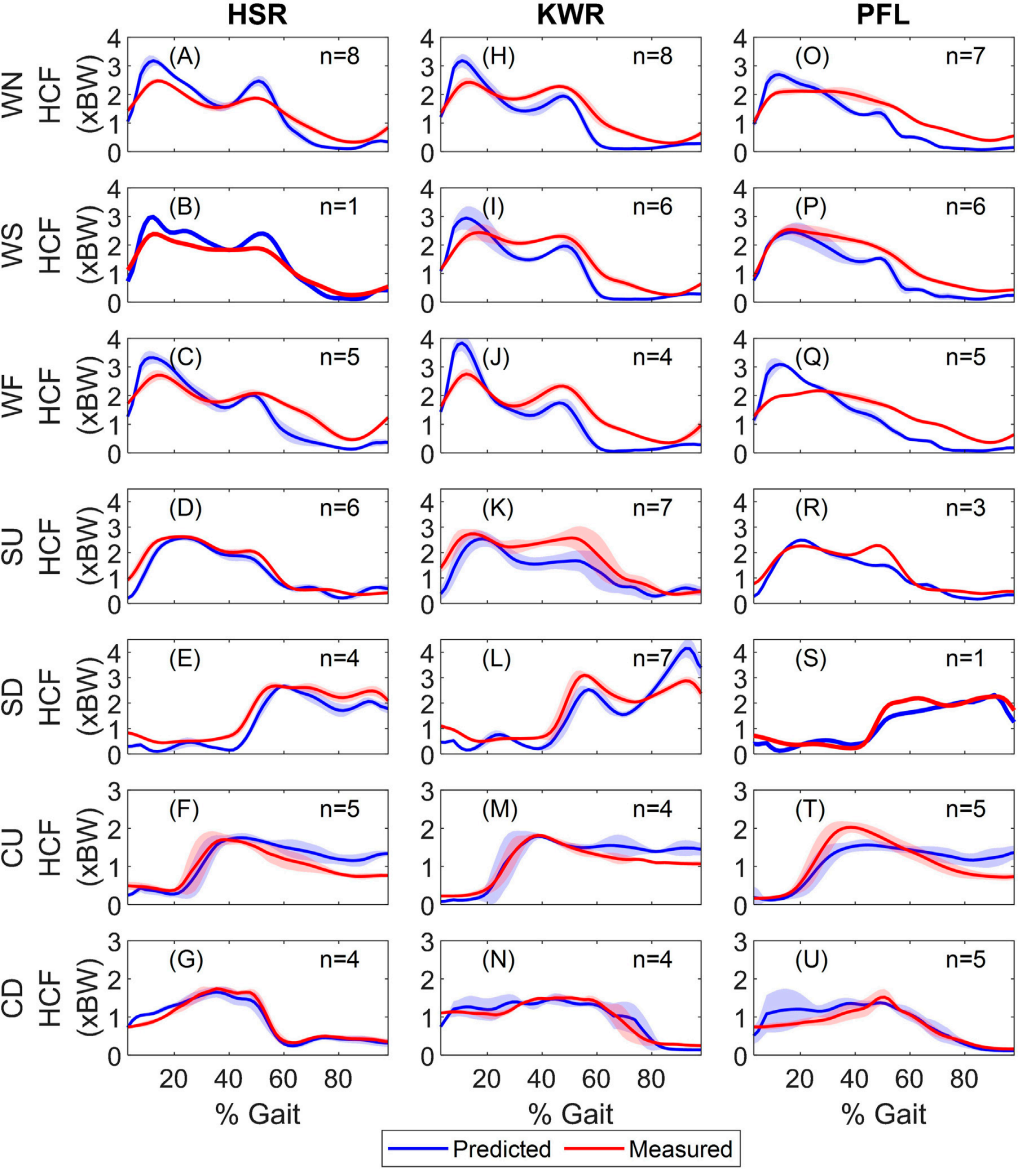
Measured and predicted hip contact forces during activities for **(A–G)** HSR, **(H–N**) KWR, and **(O–U)** PFL. The ensemble average of all trials and standard deviations are shown (n shows the number of trials for each activity). Each row shows on activity, and each column shows the results for one subject. WN, WS, WF, SU, SD, CD, and CU stand for normal walking, slow walking, fast walking, stair ascent, stair descent, sitting down and standing up, respectively. BW and HCF stand for body weight and hip contact force.

Figure 2 shows the model predicted and measured *in vivo* hip contact forces (HCF) for all subjects and Table 2. quantifies the accuracy of the predictions. In walking trials (consistently across all speeds), the model overestimated the HCF during the stance phase for subject HSR, whereas for KWR and PFL, the HCF was overestimated during the first half of the stance and was underestimated afterwards (Figure 2 and Table 2.). In terms of the timing of the peaks HCF, the errors were generally small (around 3% of gait cycle) for HSR and KWR (Table 2.; for PFL, only one experimental peak was observable, happening during early stance in slow walking, and mid-stance during the other two walking speeds, resulting in larger timing errors.) HSR showed the smallest RMSE in all walking trials compared to the other two subjects, whereas the RMSE of the predictions were consistently larger with increased walking speed for all subjects (Table 2.). *R* ^2^ was high in all walking speeds for all subjects with the lowest value (0.83) in fast walking and highest (0.98) in slow walking, both for PFL, showing good agreement between the predicted and measured HCF.

**TABLE 2:** Performance of the model for the prediction of hip contact forces. Avg and Rg stand for average (arithmetic mean) and range, and NA means Not Available. Only one trial was available for WS of HSR and SD of PFL. *R* ^2^ is the coefficient of determination, exp. peak is the experimental peak and WN, WS, WF, SU, SD, CD, and CU stand for normal walking, slow walking, fast walking, stair ascent, stair descent, sitting down and standing up, respectively.

Subject	Activity	RMSE (%BW)		*R* ^2^ (Rg)	1st peak of HCF				2nd peak of HCF			
		Avg	Rg		Magnitude error (% exp. peak)		Timing error (% gait cycle)		Magnitude error (% exp. peak)		Timing error (% gait cycle)	
					Avg	Rg	Avg	Rg	Avg	Rg	Avg	Rg
HSR	WN	42.0	35.5–50.2	0.96–0.98	29.1	25.4–34.8	1.4	0–3.0	32.1	16.4–51.0	1.2	0–2.8
	WS	30.6	NA	0.98	24.0	NA	0.1	NA	27.0	NA	2.9	NA
	WF	56.0	46.5–67.9	0.89–0.96	26.0	17.9–36.8	1.8	0.1–3.1	9.1	0.6–12.8	1.3	0.1–3.4
	SU	32.7	29.0–38.6	0.91–0.96	2.9	1.2–5.2	2.1	0.1–6.0	10.8	5.6–21.1	2.7	0–7.9
	SD	44.5	35.3–52.7	0.94–0.99	4.3	1.0–7.7	2.1	1.6–3.1	17.0	0.5–28.3	0.9	0–1.5
	CU	32.2	25.9–39.4	0.80–0.95	6.1	0.6–10.4	6.0	1.7–9.1	NA	NA	NA	NA
	CD	14.9	12.6–18.8	0.94–0.98	6.4	0.1–17.5	1.4	0–3.3	NA	NA	NA	NA
KWR	WN	50.7	43.5–61.3	0.86–0.95	30.9	25.5–38.8	2.4	0.1–5.6	15.0	1.1–25.1	1.4	0–3.2
	WS	48.2	39.2–60.9	0.86–0.94	21.0	8.8–31.5	3.7	2.9–4.4	14.0	5.2–18.5	1.5	0.1–4.7
	WF	63.2	56.1–68.7	0.84–0.92	37.4	23.1–44.9	1.8	0.1–3.0	26.9	16.1–41.5	1.1	0.1–2.7
	SU	61.1	39.8–76.4	0.83–0.94	10.6	3.1–15.0	2.9	1.5–7.7	35.0	16.3–49.4	1.5	0.1–4.7
	SD	61.8	56.6–69.1	0.85–0.91	17.3	13.6–22.9	1.8	0–3.1	44.8	31.5–58.9	0.5	0.1–1.4
	CU	25.0	22.0–30.2	0.92–0.96	4.2	1.6–10.0	1.1	0.1–2.9	NA	NA	NA	NA
	CD	19.9	13.0–30.1	0.87–0.97	10.2	4.3–17.3	4.9	1.4–10.6	NA	NA	NA	NA
PFL	WN	50.4	39.5–54.9	0.88–0.96	23.0	3.3–36.2	12.4	2.9–21.0	NA	NA	NA	NA
	WS	44.2	34.1–49.2	0.95–0.98	4.2	0.6–11.8	1.7	0–3.5	NA	NA	NA	NA
	WF	60.9	56.1–68.3	0.83–0.90	44.6	38.4–59.7	14.4	12.0–18.8	NA	NA	NA	NA
	SU	31.1	29.8–32.3	0.93–0.96	10.3	8.5–11.9	0.2	0.1–0.3	32.8	28.8–35.0	2.0	1.4–3.1
	SD	45.7	28.2–63.1	0.80–0.96	33.7	22.8–44.6	2.3	0.6–4.0	20.5	2.3–38.8	2.6	1.6–3.6
	CU	38.2	30.6–52.6	0.69–0.85	24.8	19.3–28.9	7.0	3.5–11.0	NA	NA	NA	NA
	CD	16.5	11.2–21.4	0.93–0.97	11.7	4.5–17.8	4.4	0.1–14.0	NA	NA	NA	NA

The HCF predictions were encouraging in both stair activities for HSR, while the HCF was underestimated during the late stance in stair ascending for KWR and PFL and showed large errors for KWR in stair descending. The best predictions were obtained for HSR, with the lowest average RMSE of 32.7% and 44.5% BW for stair ascent and descent, respectively, and the lowest errors in peak HCF magnitude prediction. *R* ^2^ was larger than 0.8 for all subjects in stair trials, showing that the HCF profiles were captured by the model. In terms of the timing of the HCF peaks, the model captured the timing for all subjects closely (average between 2 and 3% of the gait cycle).

In standing up experiments, the peak HCF was predicted closely for HSR and KWR, while it was underestimated for PFL (Figure 2). HCF was overestimated for all subjects during the second half of the cycle. RMSE were 32.2, 25.0, and 38.2% BW for HSR, KWR, and PFL, respectively, and *R* ^2^ ranged 0.69–0.85. The errors in magnitude and timing of the peak HCF were around 5% and less than 10% of cycle for HSR and KWR, while they were 25% and around 19% of cycle for PFL, respectively. In sitting down experiments, the predicted and measured HCF showed close agreement for all subjects. The average RMSE was between 15–20% BW and *R* ^2^ ranged 0.87–0.98. The average error in HCF peak magnitude was 6.4, 10.2, and 11.7% for HSR, KWR, and PFL, respectively (with the timing error less than 5%).

Table 3 shows the overall performance of the MSK model across the activities (to obtain these values, for each activity, the results of all trials of the three subjects were pooled. Then, average and standard deviations were obtained (Table 3).) It is evident from both Table 3 that the prediction of HCFs deteriorated with walking speed; this can be seen in RMSE, *R* ^2^, and first peak HCF errors. During the stair trials, the HCF predictions seemed to be better in stair ascent compared to descent. The former had lower average RMSE (Table 3), showing an overall better performance, and smaller errors in the prediction of both peaks of hip contact forces. The *R* ^2^ and error in timing of the peaks (average∼2%) were similar between the stair ascent and descent trials. In chair trials, the RMSE is much smaller in sitting down compared to standing up trials (31.8 ± 8.2% vs. 17.1 ± 5.0% BW), while the peak HCF predictions were similar (9.4 ± 6.2% vs. 11.7 ± 10.3%). The larger RMSE in standing up trials was the result of overestimation of the HCF during the second half of the trials (Figure 2).

**TABLE 3:** Group performance of the model for the prediction of HCF across activities. The values are shown as average (arithmetic mean) ± standard deviation. *R* ^2^ is the coefficient of determination, exp. peak is the experimental peak and WN, WS, WF, SU, SD, CD, and CU stand for normal walking, slow walking, fast walking, stair ascent, stair descent, sitting down and standing up, respectively.

		RMSE (%BW)	*R* ^2^	1st peak of HCF		2nd peak of HCF	
				Magnitude error (% exp. Peak)	Timing error (% gait cycle)	Magnitude error (% exp. Peak)	Timing error (% gait cycle)
Group average	WN	47.4 ± 6.5	0.92 ± 0.04	28.0 ± 7.4	4.9 ± 6.1	23.9 ± 13.4	1.3 ± 1.0
	WS	44.0 ± 8.5	0.94 ± 0.03	14.3 ± 10.9	2.3 ± 1.7	17.9 ± 7.2	1.9 ± 1.6
	WF	59.8 ± 7.1	0.90 ± 0.04	35.4 ± 11.0	5.7 ± 6.2	17.2 ± 11.6	1.2 ± 1.1
	SU	44.2 ± 16.8	0.92 ± 0.04	7.4 ± 4.9	2.1 ± 2.2	24.7 ± 14.6	2.1 ± 2.2
	SD	53.3 ± 12.2	0.91 ± 0.05	14.9 ± 11.3	2.0 ± 1.1	31.4 ± 17.9	0.9 ± 1.0
	CU	31.8 ± 8.2	0.86 ± 0.08	11.7 ± 10.3	4.7 ± 3.5	NA	NA
	CD	17.1 ± 5.0	0.95 ± 0.03	9.4 ± 6.2	3.6 ± 4.0	NA	NA

Across tasks, the overall prediction error (RMSE) was lowest in chair trials (i.e. sitting down and standing up); the RMSE was similar between stair and walking trials (Table 3), although the stair trials demonstrated highest variabilities. For the prediction of first peak of HCF magnitude, errors were similar for stair, chair, and slow walking trials, whereas normal and fast walking showed the highest errors. There was more variability in the prediction of the second peak of HCF in all activities, with stair trials showing highest variability (Table 3).

**FIGURE 3:**
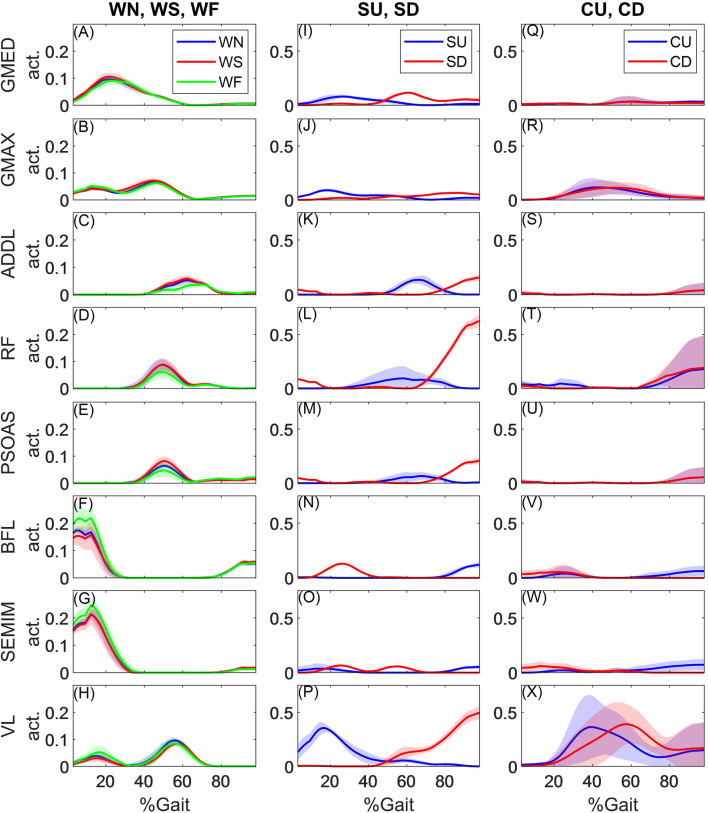
Obtained activations for hip spanning muscles act.=FiFimax during different activities for subject KWR during **(A–H)** slow, normal, and fast walking, **(I–P)** stair ascent and descent, and **(Q–X)** standing up and sitting down. The thick lines show the ensemble average of the trials, and the shaded area shows the standard deviations. GMED, GMAX, ADDL, RF, PSOAS, BFL, SEMIM, and VL stand gluteus medius, gluteus maximum, adductor longus, rectus femoris, psoas major, biceps femoris long head, semimembranosus, and vastus lateralis, respectively. For walking and stair ascending trials, the toe-off happens at around 60% of the cycle, whereas for stair descent, the cycle start with toe-off and heel strike happens at around 40% of the cycle. WN, WS, WF, SU, SD, CD, and CU, stand for normal walking, slow walking, fast walking, stair ascent, stair descent, sitting down and standing up, respectively.

Figure 3 shows representative muscle activation patterns (for KWR) during all activities. The muscle activations patterns were smooth and continuous and captured the synergistic activities among lower limb muscles. In walking trials, the activation patterns of the muscles stayed consistent across the speeds, with slight changes in the amplitudes for some muscles: biceps femoris long head (BFL), semimembranosus (SEMIM), and vastus lateralis (VL) slightly increased their peak activations with walking speed at early stance (15–20% cycle), whereas the activations of hip spanning muscles, including rectus femoris (RF), psoas major (PSOAS), and adductor longus (ADDL) reduced at late stance with walking speed. During stair ascending trials, VL, gluteus maximum (GMAX) and gluteus medius (GMED) were active at early stance to extend the knee, hip and stabilize the hip, respectively, and at late stance, RF, PSOAS and ADDL were working to swing the leg (i.e. flex the hip) up the stairs. In stair descending, at early stance (in Figure 3, heel strike happens at 40% of the cycle of stair descent trials), PSOAS and VL were active to counteract the external hip extension and knee flexion moments, respectively, while RF helped with both (as it is a biarticular muscle); during swing (from 0 to 40% of the cycle), BFL and SEMIM were active. In standing up and sitting down, VL and GMAX were the major muscles to counteract the external knee and hip flexion moments, respectively, while other hip muscles had small activations.

## 4 Discussion

The objective of this study was to utilize Freebody, a segment-based musculoskeletal model (Cleather and Bull 2015), for the prediction of hip contact forces using a novel objective function during seven common ADLs and validate its performance against the publicly available HIP98 dataset. The prediction accuracies were similar or better than the previously reported values in the literature by other MSK models (Heller et al., 2001; Stansfield et al., 2003; Modenese et al., 2011; Modenese and Phillips 2012; Moissenet et al., 2015; Mathai and Gupta 2019; Weinhandl and Bennett 2019). FreeBody predicted the hip contact forces with RMSE of 44.0 ± 8.5, 47.4 ± 6.5, and 59.8 ± 7.1% BW during slow, normal, and fast walk (Table 3). Similar values were reported by previous studies: 23.2–52.4% BW during normal walking using the LLLM (with sum of squared activation as the objective function) (Modenese et al., 2011), 50–82.2% BW (Weinhandl and Bennett 2019) and 30–86.2% BW (Mathai and Gupta 2019) for walking at different speeds using the LLLM, gait2392, ALLM, and hip2372. In terms of the peak hip contact force, FreeBody predicted the first and second peaks with average errors of 14.3% and 17.9%, 28.0% and 23.9%, and 35.4% and 17.2% during slow, normal, and fast walk, respectively. This was similar or better than other studies: 20.8% during normal walking (Modenese et al., 2011), and 18.1–49.1% at different walking speeds across 4 models (Weinhandl and Bennett 2019). Heller et al. (2001) reported a smaller value of 12% error during normal walking, however, the arithmetic mean was used to find the average error (where opposite sign cancel each other.) Stansfield et al. (2003) also reported errors (for only 2 subjects) in the ranges of 6.9–32.9%, 6.2–21.4%, and 12.2–28.8% during slow, normal, and fast walking. However, the variability of the predictions is not clear, since they only reported their average errors.

During stair ascent, FreeBody predicted the hip contact force with a RMSE of 44.2 ± 16.8% BW and peak errors of 7.4 ± 4.9% and 24.7 ± 14.6% (Table 3). Similar RMSE, ranging 20.0–61.1% BW, and 10% error in the first peak predictions were reported by Modenese et al. (2011), whereas Mathai and Gupta (2019) reported higher RMSE values, ranging 64.8–101.0% BW. Consistent with both studies, FreeBody errors mostly happened due to the underestimation of the push-off hip contact force peaks. During stair descent, FreeBody predicted the hip contact force with RMSE values of 53.3 ± 12.2% BW and higher peak errors (Table 3). Only Mathai and Gupta (2019) reported the predictions during stair descent for one subject (HSR), with RMSE ranging 31.5–83.5% BW, heavily underestimating the contact forces during push-off and the swing phase using all 4 examined MSK models. (For HSR, we found RMSE ranging 35.3–52.7% BW, showing FreeBody’s better performance).

Our segment-based model predicted the hip contact force best in standing up and sitting down experiments with RMSE of 31.8 ± 8.2% and 17.1 ± 5.0% BW, respectively, while the peak was predicted with an error of around 10% (Table 3). Two other studies examined these two activities: Mathai and Gupta (2019) reported errors of 17.7–49.8% BW in standing up and 18.9–51.1% BW in sitting down for HSR (Our RMSE for HSR was 25.9–39.4% for standing up and 12.6–18.8% for sitting down, showing better performance.). The second study by Stansfield et al. (2003) reported errors only at two specific points (at 20% and 50% of the cycle for standing up and sitting down, respectively), thus, can not be compared to our data. During standing up, FreeBody predicted prolonged, almost constant hip contact force in the second half of the cycle, whereas the measured forces decreased gradually. This was the result of co-contraction about the hip joint, among mostly PSOAS, RF (and to some extent ADDL), GMAX and the hamstrings (BFLH, and SEMIM). Although the objective function was selected to reduce hip contact forces and consequently discouraged muscle co-contractions, the required external hip flexion, hip adduction, and knee extension moments at the second half of the activity required the activations of hip extensors (GMAX and hamstrings), hip abductors (GMED), and knee flexors (again hamstrings), respectively. These, therefore, contribute to the primary plane of motion, but also give an out of plane contribution, necessitating co-contraction of other muscles (i.e. RF, PSOAS, VL) and higher predicted hip contact force. The observed prolonged activations in GMAX, hamstrings, vastii muscles, and RF have been consistent with measured EMG in standing up activity (Doorenbosch et al., 1994).

MSK anatomical datasets, representing muscle and bone geometries, are key in the accuracy of muscle and articular contact force estimation. FreeBody uses an anatomical dataset (Ding et al., 2019) obtained from high resolution MRI images of lower limbs to represent the attachment sites of 38 muscles in the lower limb, according to the methodology proposed by Klein Horsman et al., 2007. Using this MSK geometry, FreeBody has previously estimated knee contact forces during ADLs with encouraging accuracy (Ding et al., 2016). In the current study, FreeBody also predicted hip contact force during ADLs, with similar (or better) accuracy to the LLLM, which has been reported to perform better than the other anatomical models in gait2392, hip2372, and ALLM models (Mathai and Gupta 2019; Weinhandl and Bennett 2019). Similar to FreeBody, LLLM divides the muscles into several linear elements to better represent muscle function, however, gait2392, hip2372, and ALLM models are based on Delp et al. (1990) model, which is less detailed and may not sufficiently represent muscle lines of action and consequently, their moment arms, resulting in larger errors in the prediction of hip contact force.

**FIGURE 4:**
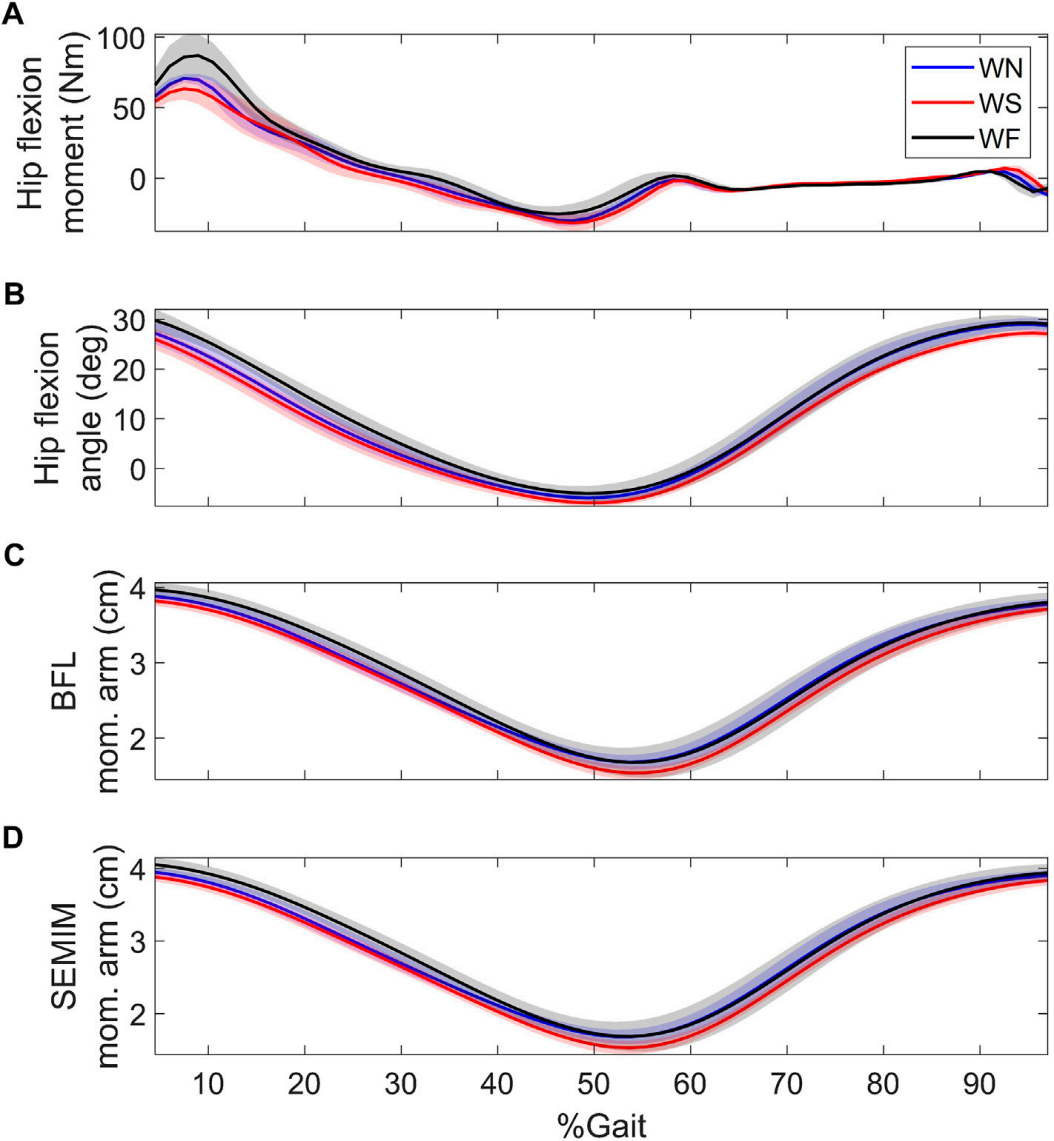
**(A)** hip flexion moment, **(B)** hip flexion angle, and sagittal plane moment arms of **(C)** BFL and **(D)** SEMIM during walking at different speeds of KWR. BFL and SEMIM stand for biceps femoris long head and semimembranosus; and WN, WS, and WF stand for normal, slow, and fast walking, respectively.

FreeBody accuracy for the prediction of hip contact force reduced with walking speed. LLLM, which uses the same MSK representation as FreeBody, also reported monotonic increase in the prediction errors with walking speed (Modenese and Phillips 2012). It was suggested that exclusion of muscle activation dynamics may have contributed to the larger errors in fast walking (Modenese and Phillips 2012). However, Weinhandl and Bennett (2019) reported that the lowest RMSE was found during normal walking, with similar RMSE during slow and fast walking; they did not account for activation dynamics either, however, they used three MSK geometries (gait2392, hip2372, or ALLM) different from LLLM or this study’s. Therefore, the MSK geometry might have played a more important role in the increased errors. Examination of the predicted hip contact forces in the current study shows substantial increase with walking speed at the loading response (i.e. around 10% of the gait cycle) for all subjects (Figure 2). For KWR (whose muscle activations are demonstrated in Figure 3), the increased contact forces during the loading response are associated with increased hamstrings forces (BFL and SEMIM). Examining external hip flexion moment shows a large increase in the moment with walking speed, while the hamstring moment arms in the sagittal plane change slightly during the loading response of different walking speeds (Figure 4); this requires larger muscle forces to counteract the hip flexion moments, which may in turn have contributed to the overestimation of muscle and hip contact force. In addition, irrespective of the choice of the objective function, there will be some errors in the prediction of hip contact forces at any walking speed due to out of plane contributions of the muscles that were not perfectly aligned to the primary plane of motion, which requires the co-activations of other muscles to stabilize the spherical hip joint and therefore results in overestimation of hip contact forces.

The choice of the objective function for the muscle force sharing greatly affects the predictions of muscle forces and consequently hip contact forces. The most commonly used objective function is the sum of normalized muscle forces (or activation) with a certain power (Crowninshield 1978). Previous studies, estimating hip contact forces from the HIP98 dataset, utilized linear (Heller et al., 2001) and double-stage linear optimization (Stansfield et al., 2003), quadratic (Mathai and Gupta 2019; Weinhandl and Bennett 2019), and powers from 1–15 (Modenese et al., 2011). An appropriate objective function should provide reasonable estimates of both muscle activation patterns and the joint contact forces. It is well documented that a linear objective function generates sparse activation patterns for muscles, favoring muscles with larger PCSA and moment arms (Crowninshield 1978; Hardt 1978; Modenese et al., 2011). Higher order objective functions produce the synergistic activities among the muscles (Anderson and Pandy 2001), however, as the power increases, higher co-contraction about the hip generates larger out of plane muscle forces and consequently result in overestimation of hip contact forces (Modenese et al., 2011). The current study used a quadratic objective function, as it was previously shown to capture muscle activation patterns and generate reasonable hip contact force estimates (Modenese et al., 2011; Modenese and Phillips 2012; Weinhandl and Bennett 2019). However, the constrained non-linear static optimization for muscle force estimation is highly dependent on initial conditions and not guaranteed to converge to a global minimum; thus, it is likely a local minimum is found, which results in higher muscle forces and overestimation of hip contact forces. A strength of the segment-based formulation of FreeBody is the simultaneous estimation of muscle and articular contact forces, which allowed us to directly include the hip contact force magnitude in the objective function to drive the muscle forces toward the true minimum, hence reducing muscle forces (while maintaining reasonable activation patterns). Using this function allowed us to consistently obtain reasonable estimates of the hip contact forces not only in walking, but across all the examined ADLs. Using similar approach, DeMers et al. (2014) showed that sum of squared muscle activations and vertical knee contact forces more accurately reproduced knee contact forces than minimizing only the former, while generating activations consistent with measured EMG of the muscles.

We qualitatively assessed the predicted muscle activation patterns against experimental EMG from healthy individuals in walking (Wootten et al., 1990; Amiri et al., 2015), stair ambulation (Lyons et al., 1983; McFadyen and Winter 1988), and standing up (Doorenbosch et al., 1994). During walking, the predicted muscle forces were consistent with the experimental EMGs. GMED and GMAX predicted forces were high during stance, with a larger peak happening at early stance, and ADDL had its peak activity at late stance immediately before toe-off, consistent with Wootten et al. (1990). Hamstring muscles (SEMIM and BFL) were highly active before heel strike and peaked immediately afterward, consistent with experimental EMGs (Wootten et al., 1990; Amiri et al., 2015). VL predicted force showed two peaks at early stance and pre-swing, whereas both single peak (Amiri et al., 2015), and double peak EMG activity have been reported for VL (Wootten et al., 1990). RF predicted forces demonstrated a late stance peak, whereas its measured EMG shows a peak at early and another in late stance (Wootten et al., 1990; Amiri et al., 2015); similar to our results, other MSK models were unable to capture the first RF peak (Modenese et al., 2011; Modenese and Phillips 2012). PSOAS forces were consistent with its activity measured intramuscular EMGs, reaching its peak before toe-off (Andersson et al., 1997).

Phasing of muscle activities have been reported to be stable (den Otter et al., 2004), while the amplitude has been reported to change with the walking speed (Murray et al., 1984; Bugle and Limbird 1987; Franz and Kram 2012). The simulated muscle forces in the current study showed similar patterns across walking speed with small changes in the amplitudes. This is probably due to small range of walking speed for the patients in HIP98 dataset (1.05, 1.15, and 1.40 m/s in slow, normal, and fast walking for KWR), whereas the change in muscle force amplitude has been reported to be significant with much higher walking speeds: 1.75 m/s (Franz and Kram 2012), 1.92 m/s (Murray et al., 1984), and 1.81 (Bugle and Limbird 1987). The most significant muscle force changes in this study seemed to happen for the hamstrings in the loading response, counteracting the higher external hip flexion moments.

During stair ascending, GMED and GMAX forces both had double peaks during stance, and SEMIM force started to increase before heel strike and reached a peak at early stance, consistent with measured EMGs (Lyons et al., 1983; McFadyen and Winter 1988). RF was active from mid-stance to mid-swing, with peak immediately after toe-off, whereas EMG measurements show two other peaks, at early stance and mid-swing (McFadyen and Winter 1988); this pattern was not captured by previous MSK models either (Modenese et al., 2011). In stair descent, consistent with EMGs (Lyons et al., 1983; McFadyen and Winter 1988), prediction for GMED showed double peak activations during stance, SEMIM showed high activities during mid-swing and early stance, and BFL had high activity only during swing. GMAX was active throughout the stance, whereas EMG shows activity mainly at early stance (McFadyen and Winter 1988). RF predicted forces were high at late stance and mid-swing, whereas the measured EMG showed another peak also at early stance, not captured by the model (McFadyen and Winter 1988).

During standing up experiments, GMAX and VL predicted forces peaked after the seat off to extend hip and the knee, respectively, and afterwards their forces gradually reduced, consistent with EMGs (Doorenbosch et al., 1994). Moreover, FreeBody predicted co-contraction of PSOAS, GMAX, hamstrings (SEMIM and BFL), RF, and VL at the end of the cycle.

There are some limitations associated with our model. We used a single objective across tasks; while this may be strength (since a single objective function provides a simpler formulation to the muscle force estimation), it may be argued that a task-appropriate objective function could have produced even better results. Second, the maximum force potential of the muscles in our model were not subject dependent, even though we modified the maximum force potential to account for the effect of hip replacement surgery and decline with age. Maximum voluntary contraction experiments could be a better identifier of maximum muscle force potentials. Third, the number of markers available with the HIP98 dataset for inverse kinematic calculations is minimal. This may have affected the obtained joint angles due to skin movement artifacts; in addition, errors in the measurement of 3D position of hip joint centre may have affected the estimated resultant hip moments, muscle moment arms about the hip, and consequently the estimated hip contact forces. Fourth, our model does not account for muscle contraction dynamics and force-length-velocity relationships (similar to other models in the literature); while the effect may be negligible during normal paced activities, it could be more important in fast walking. Fifth, we did not have access to subject-specific muscle and bone geometries, therefore, relied on linear scaling, which may generate substantial error in the prediction of the articular contact forces. Future investigation should examine non-linear scaling methods, shown to improve the MSK model predictions (Nolte et al., 2016).

In summary, this study has implemented a novel objective function in a lower limb segment-based musculoskeletal model and validated this for the prediction of hip contact forces during a large range of ADLs. The objective function incorporates the body weight normalised hip contact force that is minimised together with muscle stresses squared with an adjustable weighting factor between these two parameters. The model outputs are best in class for standing up and sitting down and compare favourably for all other activities of daily living. This new objective function addresses one of the major limitations associated with musculoskeletal models in the literature, namely the very high non-physiological predicted hip joint contact forces.

## Data Availability

Publicly available datasets were analyzed in this study. This data can be found here: https://orthoload.com/test-loads/data-collection-hip98/.
